# The Tennessee Dental Association

**Published:** 1875-10

**Authors:** 


### Proceedings of Societies.

### THE TENNESSEE DENTAL ASSOCIATION.

The ninth annual meeting of this Association was held in Nashville, June 24th and 25th, in the United States Court Rooms, corner of Cedar and Summer Sts., President E. S. Chisholm presiding and R. R. Freeman Secretary.

After transacting the usual preliminary business of the Association a lengthy paper on Extraction of Teeth, by Dr. S. P. Cutler, of Memphis, was read.

The subject was now open for discussion.

Dr. Morgan: The statement that mercury produces absorption of the alveoli and gums where no ptyalism has been produced is an assertion that can not be proven. It may be so but it is a mere inference with no data to support it.

Dr. Noel: My observation is that the demand for artificial teeth, since the introduction of Nitrous Oxide, has diminished rather than increased, and this I take to be an evidence of the increased appreciation on the part of the public of the operation of filling.

Dr. Cobb: I am satisfied that thousands of teeth have been sacrificed since the introduction of anæsthetics. I am confident that the statements of Dr. Cutler, on that point are correct. That the demand for anæsthetics is diminishing, is due more to the fact that the best men in the profession are discountenancing their use than to any ruling of fashion. I hope some action will be taken by this Association at this meeting showing its disapproval of the use of these agents.

AFTERNOON SESSION.—FIRST DAY.

The Association was called to order at 3 p. m., by the President and the discussion of Dr. Cutler's paper was resumed.

Dr. L. C. Chishplm: If there is ever a time for the extraction of a tooth it is when the nerve is dead.

Dr. Taft: This is in one sense an important subject and ought to receive the attention of every dentist. With me it is a study how to avoid extracting rather than how I can extract the greatest number of teeth. There is a class of patients who do not appreciate the worth of their teeth, and who are unwilling to pay for their treatment. In such cases nothing can be done but extract. This is one condition for extracting. Such patients are not only unwilling to pay the dentist for his efforts, but would not appreciate any thing but immediate relief of pain. Those who properly value their teeth, will secure the best service for their preservation. The dentist in operating for such a patient should only enquire; can I save the tooth. If we will endeavor to save the teeth instead of extracting, we will be surprised at the small number there will be to remove. Sometimes a month passes and I do not extract a single tooth.

Dr. Cobb; We should do every thing that can be done before resorting to the forceps. I am afraid my friend Dr. Chisholm is too heavy on dead teeth. I am afraid he'd rake them out too close. I have in my mouth a devitalized tooth that has done good service for many years and expect it to last several years longer.

Dr. Morgan: There is quite a number of persons of strumous diathesis, who can not retain devitalized teeth long, till suppuration and alveolar abscess take place. This class is largely represented by the Mulatoes; patients free from scrofula, of sound solid ossens tissues are the best subjects for the treatment of dead teeth. Patients of billious temperment with dark skin and hair and eyes are, when free from scrofula, also good subjects for these operations. Every dead tooth will ultimately give rise to trouble. They may last five ten or twenty years but trouble will come.

The subject was passed and Dr. W. H. Morgan read a lengthy paper on "Dentistry in Tennessee," in which he gave a detailed account of the rise and progress of the profession in the state.

Dr. Taft: I am glad that it has come into the mind of Dr. Morgan to write such a paper. I would be glad if every member of the society, who is aware of any historical matters that have not been made public, would contribute such facts to be added to the paper. Perhaps all present are aware that a history of dentistry in the United States is in course of preparation. It is desirable to call to aid in this work, the older members of the profession throughout the country. It is intended to use this paper in that work. There are many valuable facts liable to pass out of recollection, hence the necessity for such a work.

Dr. E. S. Chisholm read a paper on "Treatment of Exposed Pulps."

Dr. S. J. Cobb; There are many cases in which there is no chance to retain a filling after the capping of oxy-chloride has been introduced. This is one of the cases in which Dr. Cutler's plastic fillings would be required.

Dr. Taft: The ultimate preservation of a tooth depends upon the vitality of its pulp. The dentine receives its nourishment through the pulp. A gradual process of hardening is going on in the dentine from youth to old age, and a gradual diminution of the pulp canal by the elaboration of calcarious material by the pulp. Of course this hardening process is arrested as soon as the vitality of the pulp is destroyed. Devitalized teeth are undergoing deterioration from the beginning the best of them break down and ultimately crumble. Living teeth may last for forty years after being filled, but from five to ten is the average duration of pulpless teeth. Large shallow cavities or cavities on the distal side of the inferior molars such as referred to by Dr. Cobb should not deter one from undertaking to save the pulps. Don't work by guess, cut away until you can have full view of the work. In exposed pulps the formation of secondary dentine takes place oftener than we suppose and a good agent to favor this is lacto-phosphate of lime.

The Society adjourned to meet at 9 a. m. next day.

MORNING SESSION.—SECOND DAY.

The Association met according to adjournment, and was called to order by the President. The discussion of Dr. E. S. Chilholm's paper was continued.

Dr. Morgan: A number of gentlemen here were at a loss to know how Dr. Taft sees into certain distal cavities. This is very simple and plain to me. He uses the rubber dam and shuts out the saliva. I have had the same trouble but since adopting the rubber dam have had no such trouble.

Dr. Taft: I did not suppose when we were discussing this subject yesterday that there was one here so far back as to try to operate without the rubber dam. No one can operate as well without it as he can with it, no matter what he uses.

Dr. Ross: I would like to call your attention to a tooth I have, in which the nerve was found to be alive though it presented all the symptoms of a dead tooth, had even supurated. I would like to know if a tooth, with a living pulp, could have an abscess.

Dr. Taft: I have never seen a tooth, with a living pulp with an abscess.

Dr. Beach: I extracted a tooth some time since, which had given rise to trouble on account of an abscess. The nerve when examined gave all the appearance of health, but when examined with a glass a small granulation was found which had set up inflammation that had extended to the periosteum.

The subject was passed and Dr. R. R. Freeman read a paper on Dead Teeth. He said he directed his attention toward retaining the teeth in the mouth in an inoffensive condition as long as possibie. He recommended filling the roots of the bicuspids and molars with Hill's Stopping.

Dr. Beach: I have obtained good results from filling the roots of dead teeth.

Dr. Morgan: In many instances when the tooth is tender before the filling is half malleted in, all trace of soreness disappears.

Dr. Taft: This is an important subject. The dead tooth is better than no tooth at all, and therefore we must do all in our power to save it as long as possible. The idea of one course of treatment being sufficient for all teeth is nonsense. We must learn the condition of the tooth and then, guided by a sound judgment, select that which is best suited to the case· A new agent, salicylic acid, has just been introduced for the treatment of dead teeth; it is a disinfectant and antiseptic; has no odor and a sweetish taste; has been used in Germany for more than a year, but has been introduced into this country within the last few months and is giving most signal satisfaction not only in dentistry, but also in general surgery.

Dr. W. L. Dismukes read a paper on Finishing Fillings, in which he called to mind the importance of finishing fillings thoroughly. Thought thousands of fillings lost yearly on account of not being properly finished up. He exhibited a little contrivance of his own, made of emery paper varnished on the smooth side to prevent softening by moisture, cut round, the size of gun wads, to be used on a mandrel and engine.

Dr. L. C. Chisholm expressed himself pleased with it, so also did Dr. Cobb, Acree and Dr. Sandusky who thought he could improve it by doubling the paper so as to have two cutting sides instead of one.

A paper was next read by Dr. L. C. Chisholm on Mechanical Dentistry. He thought gold the best base for artificial teeth, was opposed to rubber, especially the suction plates. Thought there was less expansion and contraction in the Boston Star when vulcanized than any other, and therefore thought it the best.

Dr. Morgan: I agree with Dr. Chisholm that the teeth can be better articulated on gold than rubber, and also that gold is the best base for artificial teeth.

Dr. Acree: I never have any trouble from shrinkage and contraction such as Dr. Chisholm speaks of in his paper. I make my casts of block tin, when I have a full set to make and have never had a failure where I had good casts. I make shollow air chambers for I think thick ones a curse. I have never had any expansion, contraction or sore mouths.

Dr. Cobb; I do not like the air chamber, never saw any value in it. I removed a plate in Louisville some years ago, which had caused sore mouth, the air chamber of which was one quarter of an inch deep.

Dr. Acree: I have never seen a case of sore mouth caused by wearing a rubber plate when the plate was well fitting.

Adjourned to afternoon session.

### AFTERNOON SESSION.—SECOND DAY.

The Association met according to adjournment and resumed the discussion of Mechanical Dentistry.

Dr. Beach: Celluloid has proven a failure as a base with me. I have communicated with Mr. S. S. White's, Council, and he gave it as his opinion that attaching teeth to a plate with celluloid, would not be an infringement upon the Goodyear Dental Vulcanite Company's patent.

Dr. Taft: From twenty to thirty celluloid plates have been made in our office and not a single failure. When you have bestowed the same attention upon celluloid as upon rubber, you will meet with as much success. It is better than rubber as far as I can judge. More than fifty per cent. of all the mouths from which I have taken plates in the last two or three years have been more or less diseased. I am surprised to hear gentlemen here to-day say they have never seen a case of sore mouth caused from wearing a rubber plate. I have not used rubber as a dental plate for three or four years. Have always been opposed to its use, from its introduction.

The subject was passed. Dr. S. J. Cobb read a paper on Dental Education. Much was said concerning Dentistry as a specialty of medicine. Said he knew there were many irregularities in the profession, but did not want to be wiped out on that account, and closed by calling to mind the importance of having a dental department in connection with either the Vanderbilt University or University of Nashville.

Dr. Morgan: I have been interested in this subject for a number of years and do not think now the time for such an Institution. Not eight per cent. of all the dentists in the South have been to a dental college. Out of two hundred and fifty in this state there are not twenty graduates,

Dr. Cobb: One point in the paper I would like to hear discussed. Are we willing to abandon the name of dentistry.

Dr. Taft: I do not regard this question as important as some do. Not more than one half the physicians can stand a good examination on anatomy two years after leaving college. I do not mean to say they do not make advances. There are more hard working students in the dental profession than in the medical. The greatest demand now is for improvement in those who are already in the profession as well as for those who are coming into it.

The Association granted Dr. Taft permission to publish in the DENTAL REGISTER such of its papers as he shall deem worthy.

The election of officers for the ensuing year took place and resulted as follows:

R. R. Freeman, President.

T. E. Beach, 1st Vice President.

W. L. Dismukes, 2d Vice President.

Henry W. Morgan, Recording Secretary.

L. G. Noel, Corresponding Secretary.

A. E. Herman, Treasurer.

J. C. Ross, W. H. Morgan and S. J. Cobb Executive Committees.

Dr. E. S. Chisholm, the retiring President, read a short address thanking the Association for the courteous manner in which they had treated him.

On motion, the Association adjourned to meet in Nashville, the third Wednesday in June, 1876, unless otherwise ordered by the executive committee.

